# The neurotrophic activities of brain‐derived neurotrophic factor are potentiated by binding with apigenin, a common flavone in vegetables, in stimulating the receptor signaling

**DOI:** 10.1111/cns.14230

**Published:** 2023-04-26

**Authors:** Alex Xiong Gao, Tracy Chen‐Xi Xia, Lish Sheng‐Ying Lin, Tina Ting‐Xia Dong, Karl Wah‐Keung Tsim

**Affiliations:** ^1^ Shenzhen Key Laboratory of Edible and Medicinal Bioresources HKUST Shenzhen Research Institute Shenzhen China; ^2^ Division of Life Science, Center for Chinese Medicine and State Key Laboratory of Molecular Neuroscience The Hong Kong University of Science and Technology Hong Kong China

**Keywords:** flavonoids, neurite outgrowth, neurotrophic factors, synaptogenesis, Trk B phosphorylation

## Abstract

**Aims:**

We aimed to identify the neurotrophic activities of apigenin (4′,5,7‐trihydroxyflavone) via its coordination with brain‐derived neurotrophic factor (BNDF) and an elevated signaling of tyrosine kinase receptor B (Trk B receptor).

**Methods:**

The direct binding of apigenin to BDNF was validated by ultrafiltration and biacore assay. Neurogenesis, triggered by apigenin and/or BDNF, was determined in cultured SH‐SY5Y cells and rat cortical neurons. The amyloid‐beta (Aβ)_25‐35_‐induced cellular stress was revealed by propidium iodide staining, mitochondrial membrane potential, bioenergetic analysis, and formation of reactive oxygen species levels. Activation of Trk B signaling was tested by western blotting.

**Results:**

Apigenin and BDNF synergistically maintained the cell viability and promoted neurite outgrowth of cultured neurons. In addition, the BDNF‐induced neurogenesis of cultured neurons was markedly potentiated by applied apigenin, including the induced expressions of neurofilaments, PSD‐95 and synaptotagmin. Moreover, the synergy of apigenin and BDNF alleviated the (Aβ)_25‐35_‐induced cytotoxicity and mitochondrial dysfunction. The synergy could be accounted by phosphorylation of Trk B receptor, and which was fully blocked by a Trk inhibitor K252a.

**Conclusion:**

Apigenin potentiates the neurotrophic activities of BDNF through direct binding, which may serve as a possible treatment for its curative efficiency in neurodegenerative diseases and depression.

## Graphical abstract

In cultured human neuroblastoma SH‐SY5Y cells and rat neurons, the synergistic effects of apigenin with BDNF in stimulating the BDNF‐induced neurite outgrowth and protective effect against amyloid‐beta was demonstrated: this synergism was mediated by a direct interaction between BDNF and apigenin, through activating the signaling cascade of high‐affinity BDNF receptor, Trk B.

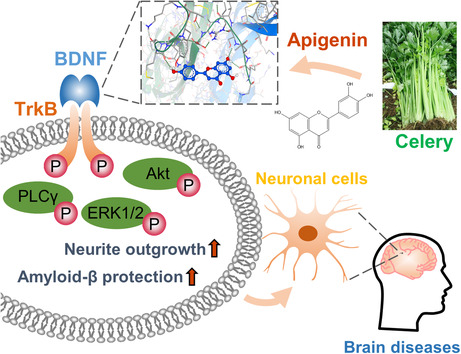

## 1 INTRODUCTION

Neurotrophic factors, e.g., nerve growth factor (NGF), brain‐derived neurotrophic factor (BDNF), neurotrophin‐3 (NT‐3), and neurotrophin‐4 (NT‐4), play vital roles in development and maintenance of neurons. Many brain diseases, e.g., Alzheimer's disease (AD), depression, are closely associated with decreased level of neurotrophic factors, particularly, the amount of BDNF.^1^, ^2^ Improving the abundance of BDNF in the brain represents a new trend in the treatment, and indeed the therapeutic outcomes are often characterized by reversing the low level of BDNF or stimulating downstream receptor signaling.^3^ However, the impermeability of blood–brain barrier (BBB) hinders the application of BDNF into the brain, and thereby small molecules were developed to mimic, or to enhance, the functions of BDNF.^4^

The binding of BDNF to its specific receptor Trk B triggered the receptor autophosphorylation and the downstream cascades, e.g., extracellular signal‐regulated kinase 1/2 (ERK1/2), protein kinase B (Akt), phospholipase C‐γ1 (PLCγ1), and cAMP response element‐binding protein (CREB). The induced signalings are accounting for the functions of BDNF in growth, differentiation, survival, plasticity of neurons. Several neuronal cell lines, e.g., NT‐2/D1, SH‐SY5Y, PC12 cells being transfected with cDNA encoding Trk receptor, have been utilized to demonstrate the functionality of BDNF.^5^, ^6^, ^7^ In particular, the SH‐SY5Y cell is the most common cell model to test the functions of BDNF.^8^ The primitive SH‐SY5Y cells do not respond to BDNF, while the treatment of retinoid acid can induce the cells to express Trk B, as a result the cell is being responded to BDNF challenge. In nonserum environment, the application of BDNF nourishes the cells to maintain cell viability and to protrude neurite forming elongated and intersected processes. Moreover, BDNF could be applied to facilitate neurite outgrowth of cultured cortex and hippocampal neurons, having the sensitivity higher than that of NGF. BDNF has also been shown to protect neurons from being attacked by amyloid beta (Aβ), the representative biomarker of AD.^9^ Besides, glial cells, both astrocytes and microglia, showed high sensitivity to the challenge of BDNF.^10^, ^11^ Therefore, the functions of BDNF‐like drug candidates could be directly evaluated via those cell models.

Flavonoid is a class of compounds commonly found in fruits and vegetables, showing large varieties of pharmacological activities, including beneficial effects to the brain.^12^ Apigenin (4′,5,7‐trihydroxyflavone) is one of the most representative compounds belonging to flavone subclass of flavonoids. The neuroprotective and neurotrophic factor‐like functions of apigenin have been reported,^13^ even though the exact upstream target(s) has not been well identified. One of the possible mechanisms is the direct binding of small molecules onto the protein/peptide ligands, e.g., growth factors. This notion is fully supported by identification of VEGF binding herbal compounds. Kaempferol, a major flavonoid in Ginkgo leaf and other edible plants, is being shown binding onto the heparin‐binding domain of VEGF, and thus this binding potentiated the angiogenic functions of VEGF in various models.^14^ In contrast, resveratrol and its natural analogs bound VEGF in suppressing the angiogenic functions.^15^, ^16^ Luteolin, a flavonoid in fruit and vegetable, has neurotrophic functions by serving an activator and a binding partner of NGF.^17^ Under this scenario, we hypothesized that the neurotropic roles of apigenin could be mediated by direct binding to BDNF, as such to potentiate its receptor signaling.

In search for active phytochemical, the binding of apigenin to BDNF was evidenced by molecular docking, ultrafiltration assay, and biacore assay. The roles of apigenin in facilitating the BDNF‐induced neurogenesis were demonstrated in cultured SH‐SY5Y cells and rat cortical neurons. Meanwhile, the coordination of apigenin and BDNF in reducing the Aβ_25‐35_‐induced cell toxicity and mitochondrial dysfunction was determined. Coinciding with the enhanced BDNF function, apigenin potentiated the BDNF‐triggered Trk B receptor for its autophosphorylation and dimerization, thereby activating the receptor signaling cascades. This study provides a new rationale in developing apigenin as nutraceutical products to treat neurotrophin deficiency‐associated neurodegenerative diseases and depression.

## 2 MATERIALS AND METHODS

### 2.1 Chemicals

Apigenin, >98% purity determined by HPLC, was obtained from the Yuanye Biotechnology. Human BDNF was purchased from Alomone Labs. Amyloid‐beta (Aβ)_25‐35_, retinoid acid, oligomycin, carbonyl cyanide 4‐(trifluoromethoxy)phenylhydrazone (FCCP), and rotenone/antimycin A were purchased from Sigma‐Aldrich. Aβ_25‐35_ was dissolved in Milli‐Q at 2 mM and incubated at 37°C for 2 days before being applied to the cultures. An inhibitor of TrkB, k252a, was purchased from Cell Signaling Technology. Probes of propidium Iodide (PI), tetramethylrhodamine ethyl ester perchlorate (TMRE) and dihydroethidium (DHE) were obtained from MedChemExpress. MitoSOX and all the culture reagents were from Thermo Fisher Scientific.

### 2.2 Molecular docking

Chemical structures of molecules were downloaded from PubChem, and the protein structure of BNDF protein was downloaded from Protein Data Bank (PDB ID:1B8M). Virtual screening was performed on SEESAR software (https://www.biosolveit.de/), as below (i) The binding site was determined according to the residues forming the identified druggable pocket. Ligand binding states, including protonation and tautomeric forms, were subsequently evaluated using ProToss method to generate the most accessible hydrogen network. (ii) Docking modulation was performed using the “Compute LeadIT Docking” mode in the FlexX algorithm; 10 binding conformations for each ligand were generated. (iii) The binding energy (i.e., ∆G) and estimated HYDE affinity (KiHYDE) for each ligand pose were calculated using the “Assess Affinity with HYDE in SEESAR” mode in HYDE rescoring function.^18^

### 2.3 Ultrafiltration‐based affinity assay

Ultrafiltration assay was performed according to the modified procedure.^19^ Briefly, apigenin at 2 μM was incubated with 2 nM BDNF in cold room for 2 h with a slow rotation. Then, the solution was loaded onto a 500‐μL ultrafiltration tube (2000 MW cutoff; Sartorius Stedim Biotech) to retain BDNF on the ultrafiltration membrane. After three times of washing and centrifugation, the unfiltered part was precipitated by acetonitrile, and the supernatant was detected by HPLC‐MS/MS. Chromatography separations were conducted on a ZORBAX XDB‐C18 column (4.6 × 50 mm, 1.8 μm; Agilent). Solvents used were as follows: solvent A, 0.1% diluted aqueous formic acid; and solvent B, 0.1% formic acid in acetonitrile. The elution procedure was set as follows: solvent B increased from 2% to 100% at 0 to 20 min; 100% to 2% at 20 to 20.1 min; 2% isocratic elution at 20.1–25 min. The injection volume was 5 μL, and the flow rate was optimized at 0.3 mL/min. Agilent QQQ (6410A) equipped with an ESI ion source was utilized for chemical ionization. The capillary voltage on ESI was 4000 V; The drying gas temperature and gas flow were 325°C and 10 L/min respectively; Nebulizer pressure used was 35 psi and the fragmentor was set as 200. The parent/product ion pair for apigenin is 271.1/153, with a collision energy of 33.

### 2.4 Surface plasmon resonance assay

The binding affinity between BDNF and apigenin was assayed on a Biacore T100 (GE Healthcare). Briefly, BDNF (20 μg/mL in water) was sufficiently captured by a CM5 sensor chip when flowing across the surface of chip. The coupling buffer was 10 mM NaAC, 150 mM NaCl, pH at 5.5. Apigenin was diluted into series concentrations of the running buffer (20 mM PBS at pH 7.4, with 2.7 mM KCl, 137 mM NaCl, 0.05% surfactant P20, and 2% DMSO) and then sequentially flowed across the chip for analysis. The regeneration was performed with 10 mM glycine–HCl solution, pH 2.0. The increased response resonance units (RUs) were instantly recorded for each apigenin solution after attaining a steady state. Data were fitted to a steady affinity model in Biacore T100 software, with the calculated KD value.

### 2.5 Cell culture

Human neuroblastoma, SH‐SY5Y cells (ATCC 1721), were cultured in a humidified incubator (37°C; 5% CO_2_) with DMEM medium containing 15% FBS. SH‐SY5Y cells were seeded onto 6‐well plate (2 × 10^4^ cells/mL) and incubated for 24 h. The medium was replaced by 2% FBS DMEM supplemented with 10 μM retinoid acid. Following 4 days of pretreatment with retinoid acid (replace with fresh medium in between), cultures were changed into nonserum DMEM medium with BDNF, or apigenin, for another 3 days of culture. Cells were fixed by 4% paraformaldehyde prior to being photographed via a phase‐contrast microscope (Carl Zeiss). A cell/cell clump with at least one visible neurite connecting to neighbored cells was counted as the cell with neurites. The percentage of cells with connected neurites was calculated according to >100 cells from 5 randomly selected imaging fields for each well. To test the neurotrophic function of apigenin and BDNF in maintaining the cell viability in nonserum stress, cells were seeded in a 96‐well plate at a density of 5 × 10^4^ cells/mL, followed by 4 days of retinoid acid pretreatment, as well as 2 days of apigenin/BDNF treatment. The Aβ_25‐35_ protective effect of apigenin/BDNF was carried out in DMEM medium with 2% FBS. One hour after BDNF/apigenin treatment, Aβ_25‐35_ was applied to the cultures for 24 h.

Rat hippocampal and cortical neurons were prepared as described.^20^ The tissue was dissected from E18 Sprague Dawley (SD) rat embryo and digested with trypsin into cell suspensions. FBS at 10% with DMEM was used as seeding medium, and the cells were seeded onto poly‐L‐lysine coated multi‐well plate with or without coverslip. Four hours after plating, the cultures were replaced with Neurobasal plus 1 × B27 supplement (Thermo Fisher Scientific). Half of the medium was changed every week for sustained cultivation. BDNF and apigenin were added onto DIV4 or DIV11 cultures at indicated time. One hour after BDNF/apigenin treatment, Aβ_25‐35_ was applied to the cultures for 24 h.

### 2.6 Cell viability assay

The cell viability was evaluated by 3‐(4,5‐dimethylthiazol‐2‐yl)‐2,5‐diphenyl tetrazolium bromide (MTT) methods. Cells were cultured in 96‐well plate for indicated time and drug treatment. MTT was diluted in basal DMEM at the final concentration of 0.5 mg/mL, which was then applied to cells by replacing the prior culture medium. After 4 h of incubation, DMSO was used to lyse the cells and absorbance at 570 nm was recorded by a microplate reader. The reading was adjusted by deducting the value of blank well, and then normalized by the untreated control group.

### 2.7 Transfection of DNA constructs

For SH‐SY5Y cells, the transfection of pNF200‐Luc constructs was performed by jetPRIME (Polyplus) reagent on the fourth day of retinoic acid induction. Subsequent to 6 h of transfection, the medium was replaced by 2% DMEM with apigenin/BDNF for 2 days of treatment. Lipofectamine 3000 (Thermo Fisher Scientific) was used for the transfection of cultured neurons. Six hours after transfection, cells were exposed to apigenin and BDNF for 2 days. Luciferase assay was performed by a commercial kit (Thermo Fisher Scientific), and the intensity was detected in a luminometer (Promega). The value of each sample was normalized by protein concentration.

### 2.8 PI and immunofluorescence staining

Upon 24 h of Aβ_25‐35_ treatment, the cells were washed once with PBS, and stained with Hoechst 33342 (5 μg/mL) for 10 min, followed by 15 min of PI (2 μg/mL) staining. Images were taken via Celldiscoverer7 (Carl Zeiss), and the density of PI‐positive cells was calculated for at least 5 fields. In immunofluorescent analysis, the cells cultured on coverslip were fixed with 4% PFA and were blocked by PBST (PBS + 0.2% Tween 20) with 4% BSA and 0.3% Triton X‐100. After washing, the primary antibodies, PSD95 and MAP2 (Cell Signaling Technology) diluted in 1% BSA in PBST, were employed at 4°C. Following overnight incubation, the secondary antibody conjugated with Alexa 594 (Cell Signaling Technology) was used to probe the protein targets. After loading with DAPI in mounting medium, the images for MAP2 and PSD95 stainings were taken via Celldiscoverer7 and LSM980 confocal microscope (Carl Zeiss), respectively.

### 2.9 Mitochondrial membrane potential (MMP) and reactive oxygen species (ROS)

To determine the change of mitochondrial membrane potential (MMP) in cultured SH‐SY5Y cells and cortical neurons after Aβ_25‐35_ treatment, JC‐1 probe (1 μg/mL) was dissolved in basal DMEM and loaded to PBS‐rinsed culture for 30 min of staining at 37°C incubators. Following another PBS washing step, the fluorescence intensity ratio of red (Ex: 525 Em: 595) to green (Ex: 490 Em: 530) was tested via FlexStation microplate reader (Molecular Devices, San Jose, CA). Dihydroethidium (DHE) and MitoSOX were used to label the intracellular ROS and mitochondrial ROS, respectively, in SH‐SY5Y cells. Following 24 h of Aβ_25‐35_ treatment, the cultures were twice washed with PBS, and then applied with 20 μM DHE or 5 μM MitoSOX dissolved in basal DMEM. After 30 min of incubation, cells were washed, and images were taken via Celldiscoverer7. The density of DHE‐positive cells, as well as the averaged fluorescent intensity of MitoSOX, were analyzed.

### 2.10 Mitochondrial bioenergetics analysis

Bioenergetic features of the SH‐SY5Y cells were measured by using Seahorse XFp Extracellular Flux Analyzer (Agilent). Briefly, the cells were seeded in Seahorse XFp‐cell culture miniplate (Agilent), and then applied with apigenin/BDNF preincubation for 1 h, as well as Aβ_25‐35_ challenge for 24 h. Afterward, the medium was gently changed with Agilent XF seahorse DMEM and incubated for 2 h in 37°C incubator without CO_2_. Then, the mini assay plate was inserted into the Seahorse machine with the help of a Seahorse XFp‐extracellular flux cartridge (Agilent) that was preloaded with mitochondrial uncouplers and inhibitors, i.e., oligomycin (complex V inhibitor), FCCP (mitochondrial uncoupler) and a mixture of antimycin A/rotenone (complex I and complex III inhibitor, respectively). After being added to the cells, the final concentrations of those inhibitors were 1, 3, and 1 μM, respectively. The real‐time oxygen consumption rate (OCR) was collected for baseline and after sequential injection of three mitochondrial inhibitors. In total, 12‐time points were recorded, and the result of each well was normalized by protein concentration determined by the Bradford method.

### 2.11 Western blotting

The cells were collected and lysed on ice with lysis buffer (Cell Signaling Technology), supplemented with 100 mM PMSF. After configuration, the supernatant was mixed with a 5 × SDS‐PAGE gel loading buffer, and the protein concentration of each sample was determined. An 8% gel was applied in SDS electrophoresis with loading amount of 25 μg per well. After gel separation, proteins were electrically transferred to the nitrocellulose membrane. The unspecific sites were blocked with 2.5% BSA, and the primary antibody was employed overnight at 4°C. Monoclonal HRP‐conjugated secondary antibody was employed to detect the blots and was visualized by a Pierce ECL substrate (Thermo Fisher Scientific). For the quantification of phosphorylated targets. Apigenin and BDNF was added to the cultures for 15 min of incubation. The supernatant was then immediately aspirated, and the cells were digested with 2 × direct lysis buffer (125 mM Tris–HCl, pH 6.8, 4% SDS, 20% glycerol, 2% 2‐mercaptoethanol). The primary antibodies against β‐actin, neurofilament‐68 (NF68), neurofilament‐160 (NF160), neurofilament‐200 (NF200), PSD‐95, synaptotagmin, Trk B, phospho‐p44/42 MAPK (ERK1/2) (Thr202/Tyr204), p44/42 MAPK (ERK1/2), phospho‐Akt (Ser473), Akt, PLCγ1, phospho‐PLCγ1 (Tyr783), phospho‐CREB (Ser133), and CREB were purchased from Cell Signaling Technology. Phospho‐Trk B (Tyr705) was obtained from Sigma‐Aldrich. The protein bands were photographed under the ChemiDoc system (Bio‐Rad), and the intensities were analyzed by Image J.

### 2.12 Statistics and other analysis

The amount of protein was determined by the Bradford protein assay. All the statistical analyses were performed in GraphPad prism 8.00 software. The Shapiro–Wilk test was employed to assess the normality for all the data. The two‐tailed unpaired t‐tests was used for comparison of two groups. Difference among multiple groups was evaluated by One‐Way ANOVA with Bonferroni post‐hoc test. When data failed to pass the normality test, the Mann–Whitney and the Kruskal–Wallis tests were used as non‐parametric equivalents. Statistical significance was determined as (*) when *p* < 0.05.

## 3 RESULTS

### 3.1 Apigenin binds with BDNF

**FIGURE 1:**
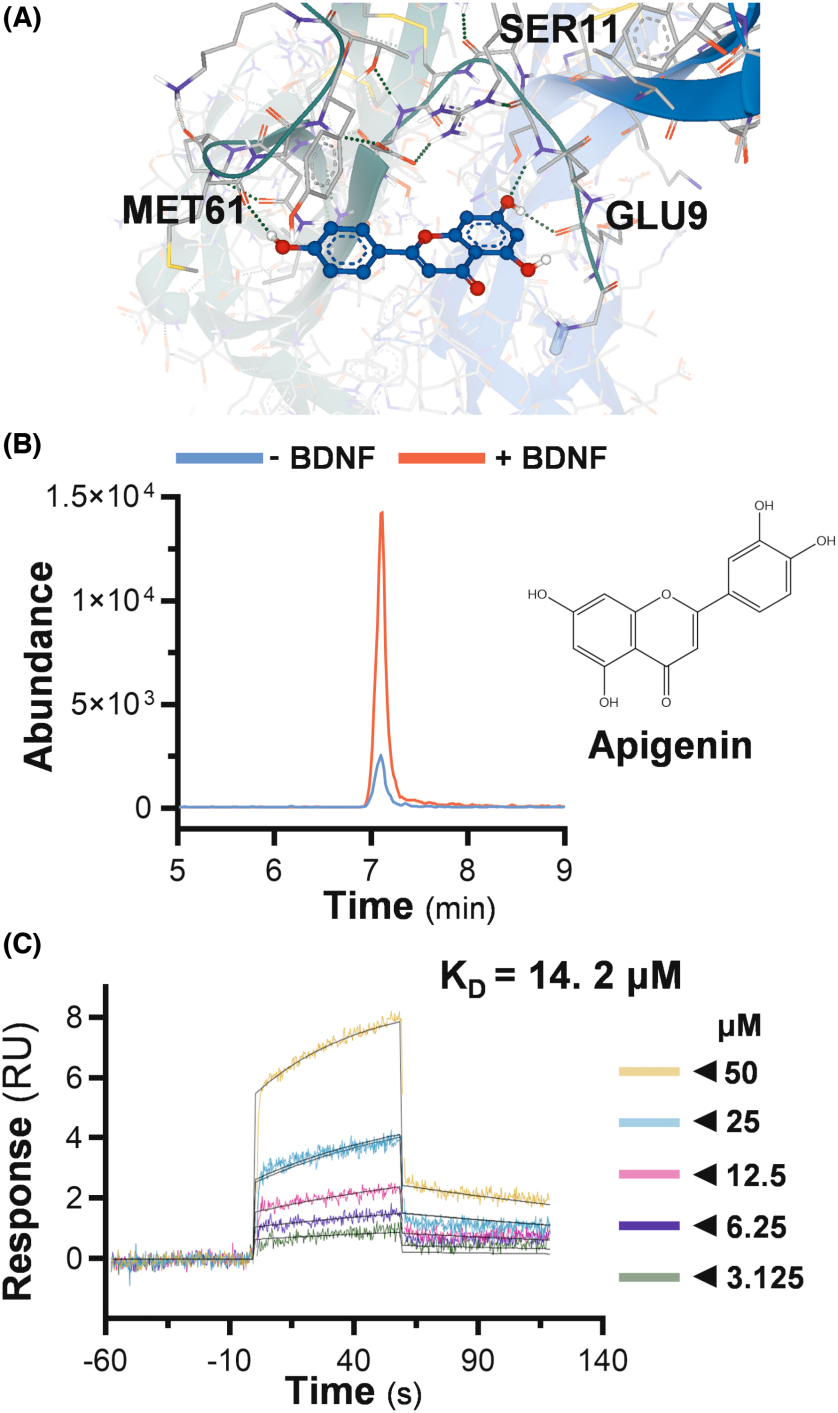
Apigenin binds with BDNF. (A) Structures of BDNF and apigenin were retrieved from PDB (1B8M) and PubChem database, respectively. The molecular docking was performed in SEESAR software, and the visualization of apigenin‐BDNF interaction was shown. (B) The binding of BDNF and apigenin was validated in an ultrafiltration‐based assay. The abundance of apigenin in the presence or absence of BDNF was tested via HPLC–MS/MS. (C) Biacore T100 platform was employed to determine the binding affinity between BDNF and apigenin. The real‐time RU change in response to different concentrations of apigenin was shown. RU values in steady‐state were identified and fitted to a steady affinity model in Biacore T100 evaluation software.

Flavonoids have been shown binding to NGF, and thereafter which potentiated the neurotrophic functions of NGF.^17^, ^21^ Considering the structure similarity among the neurotrophic factors, the flavonoids could interact with BDNF and thereby change the function of BDNF. Having this hypothesis, a molecular docking study was performed among the common flavonoids to determine their potential binding affinities to BDNF. As a result, apigenin was shown to have good binding energy at −10.9 kJ/mol (Figure 1A), and thus which was chosen for further exploration.

An ultrafiltration‐based approach was employed to validate the direct binding between apigenin and BDNF, where the apigenin was incubated with or without BDNF, followed by ultracentrifugation in a filter tube. Apigenin showed higher abundance in the presence of BDNF, i.e., retaining BDNF in the filter membrane (Figure 1B). Moreover, a surface plasmon resonance assay was performed with Biacore equipment, showing the binding in a dose‐dependent manner with a calculated *K*_*D*_ value at ~14.2 μM. These results indicated a direct binding of apigenin to BDNF (Figure 1C).

### 3.2 Apigenin enhances neurotrophic activities of BDNF in SH‐SY5Y cells

**FIGURE 2:**
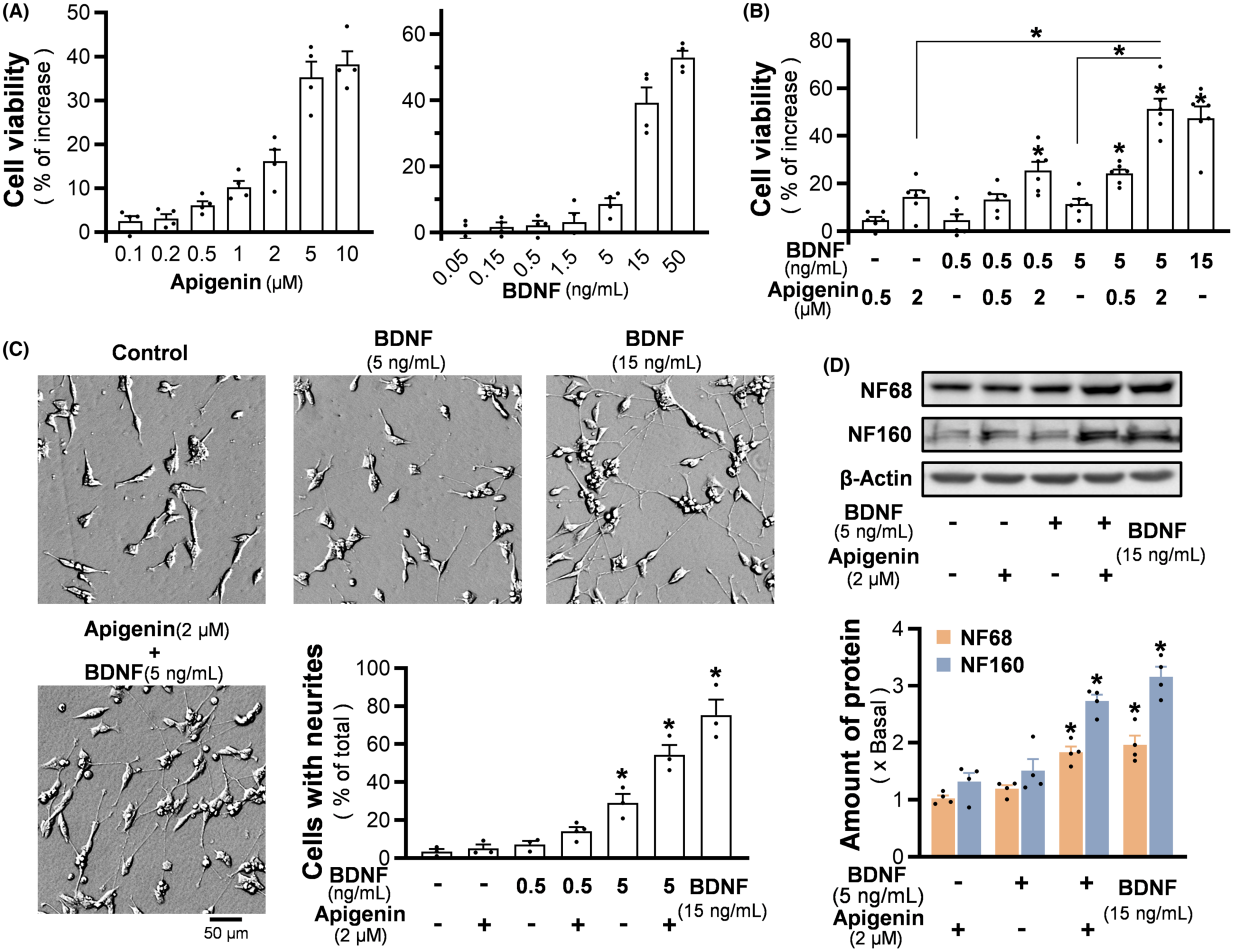
Synergy of apigenin/BDNF promotes viability and neurite outgrowth of SH‐SY5Y cells. (A) After 4 days of exposure to retinoic acid, the medium was changed into serum‐free DMEM with apigenin (left), or BDNF (right), for 2 days. The cell viability was measured by MTT assay. (B) Apigenin and BDNF were co‐applied for 2 days in serum‐starved cultures. BDNF at 15 ng/mL severed as a positive control. (C) After retinoid acid induction, BDNF and/or apigenin were applied to SH‐SY5Y cells in serum‐free DMEM medium for 3 days. The representative images were shown (left), and the percentage of total cells with connected neurites was analyzed (bottom right). (D) After the treatment as in (C), cells were collected for western blot analysis to determine the expression of NF68 (~68 kDa) and NF160 (~160 kDa). The amount of β‐actin (~45 kDa) was used as a loading control. Values are expressed as percentage of increase in (A, B), or as percentage of total number of cells in (C), or as the fold of change in (D), in mean ± SEM, *n* = 3–6. (*) *p* < 0.05.

Apigenin, or BDNF, was applied onto the retinoic acid pre‐treated SH‐SY5Y cells under basal DMEM medium: both agents effectively improved the cell viability, attaining a maximal of ~50% of increase, as compared with the untreated control group (Figure 2A). Apigenin at 0.5 and 2 μM, or BDNF at 0.5 and 5 ng/mL, only showed minor increase of cell viability: these concentrations were chosen to evaluate the possible synergy by the co‐application. The combined apigenin and BDNF nurtured the cells better than being applied alone. Especially, 2 μM apigenin and 5 ng/mL BDNF together exhibited maximal trophic function, as compared to the high dose of BDNF at 15 ng/mL (Figure 2B). In addition, BDNF, dose‐dependently, stimulated the neurite outgrowth of SH‐SY5Y cells, whereas apigenin at any concentration was not able to exert a noticeable effect (data not shown). Apigenin at 2 μM potentiated the BDNF‐induced neuronal differentiation, i.e., the percentage of connected cells with neurite was increased significantly (Figure 2C). Meanwhile, this synergy was revealed by the expression of differentiation marker, neurofilament‐68 (NF68), neurofilament‐160 (NF160), and neurofilament‐200 (NF200) (Figures 2D, S1). The induction of protein expression was more robust in the level of NF‐160. These results suggested that apigenin could work synergistically with BDNF, particularly at low‐concentration range, in inducing the differentiation of SH‐SY5Y cells.

### 3.3 Apigenin promotes BDNF‐induced neurogenesis in cultured rat neurons

**FIGURE 3:**
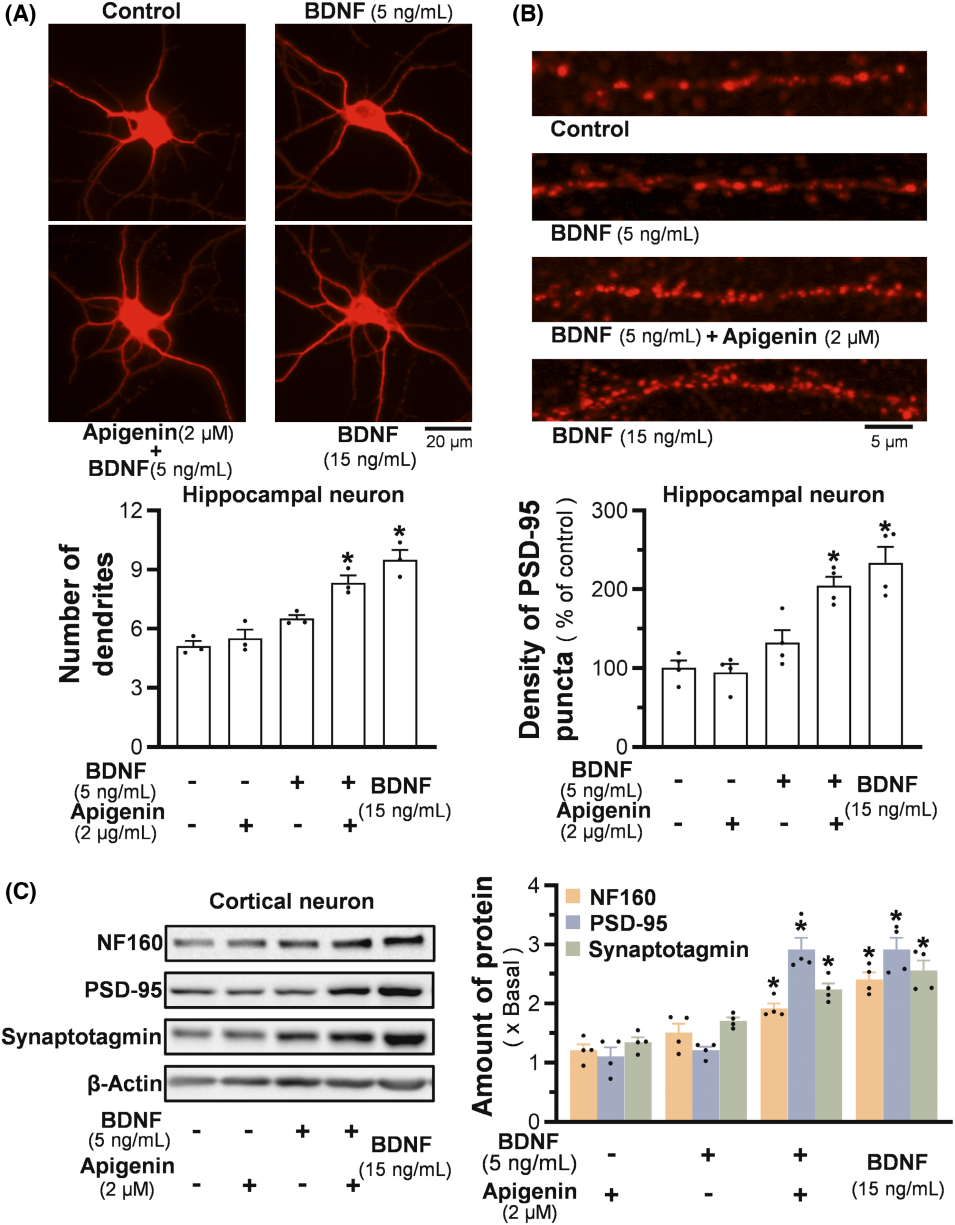
Apigenin/BDNF synergistically promotes neurogenesis in cultured neurons. (A) Hippocampus neurons were treated with BDNF and apigenin on DIV4 for 3 days. The differentiation of cell was defined by MAP2 immunofluorescent staining (upper), and the averaged number of primary dendrites was analyzed (lower). BDNF at 15 ng/mL severed as a positive control. (B) Hippocampal neurons were treated with BDNF and/or apigenin on DIV11 for 3 days. The degree of synaptic function was characterized by PSD‐95 staining (upper). The density of PSD95 puncta was quantified (lower). (C) Cortical neurons were treated with apigenin/BDNF on DIV4 for 3 days, then the cells were harvested for the determination of expressions of NF160 (~160 kDa), synaptotagmin (~65 kDa), and PSD95 (~95 kDa) (left). Protein quantification (right) was in reference to β‐actin (~45 kDa). Values are presented as averaged dendrites number (A), or density of PSD95 puncta (B), or the fold of change to the control (C), in mean ± SEM, *n* = 3–4. (*) *p* < 0.05.

In cultured hippocampal neurons, the neurogenesis status was manifested by immunostaining of MAP2 protein (Figure 3A upper panel). An increase of dendrites was demonstrated after the cells being incubated with BDNF at 15 ng/mL for 3 days. BDNF at 5 ng/mL plus 2 μM apigenin induced the cells to better differentiated at ~8 dendrites per cell (Figure 3A lower panel). In comparison, BDNF at 5 ng/mL alone exerted a minor stimulation, while the effect from 2 μM apigenin was insignificant (Figure 3A lower panel). Similar scenario was revealed for the synaptic differentiation of hippocampal neurons, as determined by PSD95 staining: the co‐treatment of BDNF at 5 ng/mL with apigenin showed significant induction on the number of synaptic buttons (Figure 3B). In parallel, the expressions of neurogenic biomarkers, i.e., NF160, synaptotagmin and PSD95, were induced markedly in the co‐treatment, showing a synergistic action (Figure 3C).

### 3.4 Apigenin and BDNF synergistically protect the Aβ‐induced cytotoxicity

**FIGURE 4:**
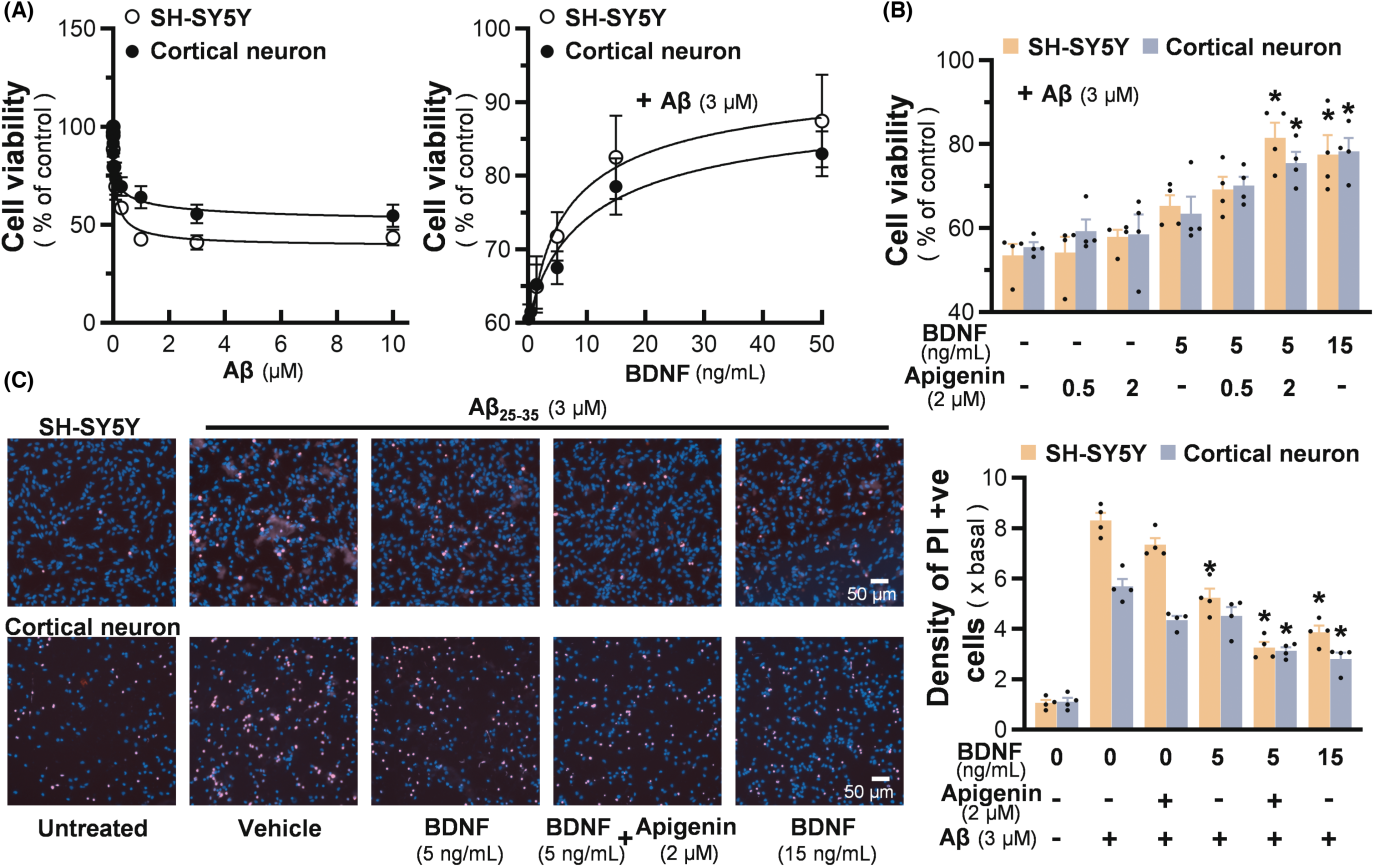
Apigenin/BDNF synergistically protects Aβ‐induced toxicity in neuronal cells. (A) Different doses of Aβ_25‐35_ were applied onto retinoic acid pre‐treated SH‐SY5Y cells, or cultured cortical neurons at DIV4, for 24 h (left). Cultures were pretreated with different concentrations of BDNF for one hour, followed by 24 h of Aβ exposure at 3 μM (right). Cell viability was measured by MTT assay. (B) BDNF and apigenin were co‐applied to cultures from Aβ_25‐35_ at 3 μM. Cell viability was measured by MTT assay. (C) The protective effect of apigenin and BDNF against Aβ_25‐35_ was tested by the staining of PI and Hoechst 33342 (left). The density of PI‐positive cells was quantified (right). Values are expressed as percentage of control, or the fold of change, compared to the control, in mean ± SEM, *n* = 4–6. (*) *p* < 0.05 against Aβ‐treated control.

Apigenin and BDNF were employed together to alleviate the Aβ‐mediated cytotoxicity in cultured SH‐SY5Y cells being pretreated with retinoic acid, as well as in cortical neurons. Upon Aβ_25‐35_ treatment, the cell viability declined in a dose‐dependent manner in both cultures (Figure 4A left panel). Three μM of Aβ having induction of ~50% cell death was selected to validate the protective role of apigenin and BDNF (Figure 4A right panel). The treatment of BDNF restored the viabilities of SH‐SY5Y and cortical neurons, as damaged by Aβ_25‐35_, with a dose‐dependent manner, having the maximal protection at a concentration higher than 15 ng/mL of BDNF (Figure 4A right panel). Apigenin enhanced the protective efficiency of 5 ng/mL BDNF to a maximum, i.e., the cell viability was restored to ~80%. Apigenin alone did not show apparent protective activity at concentration up to 2 μM (Figure 4B). Furthermore, the PI staining was carried out to probe the dead cells. In both cultures, the co‐treatment of apigenin with BDNF showed better protection than applied alone in reducing the density of PI‐positive cells (Figure 4C).

### 3.5 Apigenin and BDNF synergistically alleviate the Aβ‐induced mitochondrial stress

**FIGURE 5:**
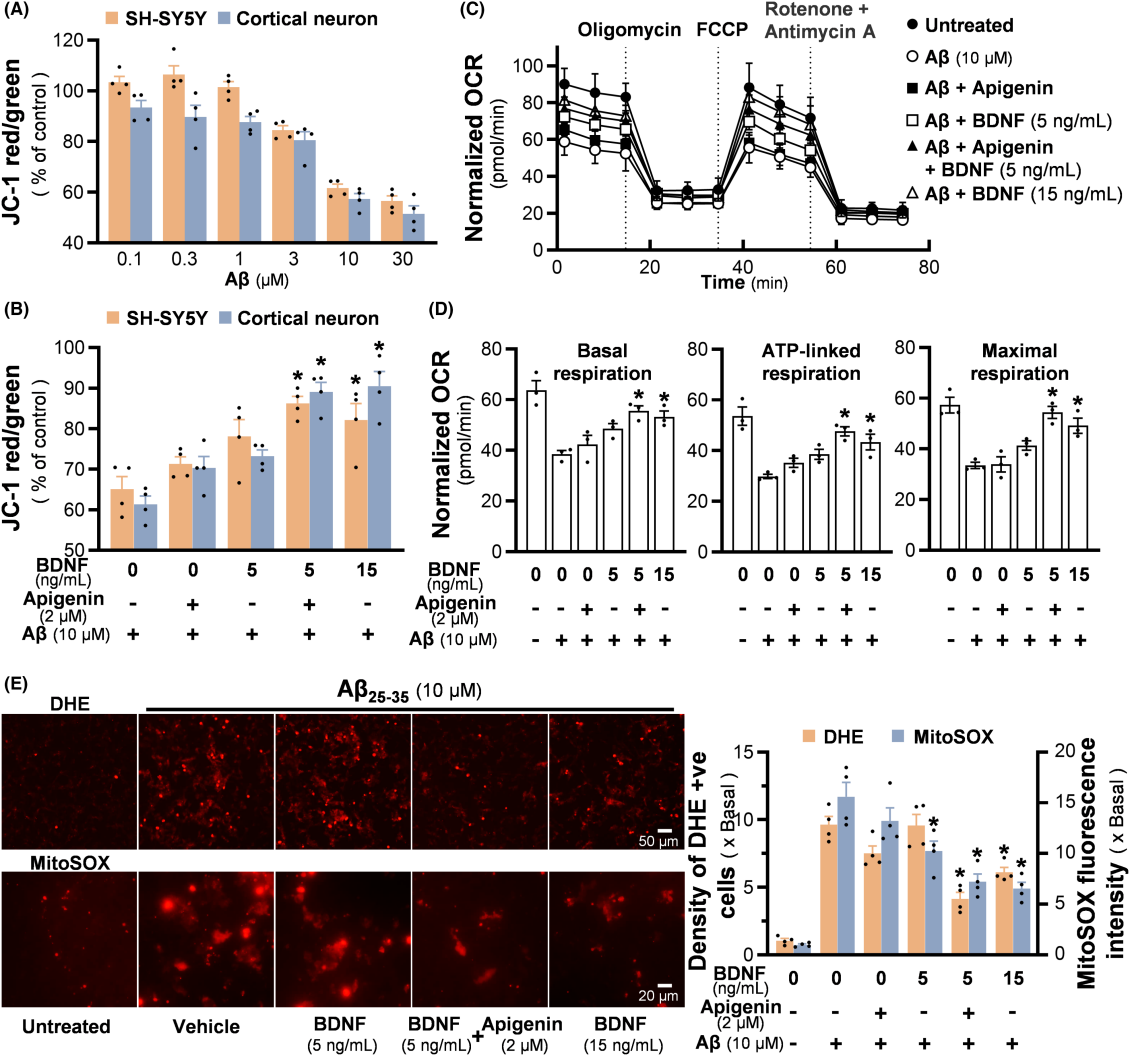
Synergy of apigenin/BDNF protects Aβ‐induced mitochondrial stress. (A) Different doses of Aβ_25‐35_ were applied onto retinoic acid pre‐treated SH‐SY5Y cells, or cultured cortical neurons for 24 h. MMP was analyzed by a fluorescent microplate reader via JC‐1 method. (B) Cultures were pretreated with apigenin and/or BDNF for one hour, followed by 24 h of Aβ exposure. Then, the protection of apigenin/BDNF on Aβ‐induced MMP loss was tested. (C) Following apigenin/BDNF pre‐incubation and 24 h of Aβ exposure, mitochondrial bioenergetic status of SH‐SY5Y cells was determined by Seahorse Analyzer. Three respiratory chain inhibitors were given to the culture at indicated time points, and the instant OCR was recorded three times after each action. The normalized OCR change with time was shown. (D) Three mitochondrial bioenergetic parameters, i.e., basal respiration, ATP‐linked respiration, and maximal respiration, were quantified by analyzing the OCR in (C). (E) The alleviation of apigenin and BDNF on Aβ_25‐35_‐induced intracellular and mitochondrial superoxide were tested via DHE and MitoSOX staining (left). The density of DHE‐positive cells and mean MitoSOX fluorescence intensity were quantified (right). Values are expressed as percentage of control, compared to the untreated group, or as normalized OCR, or as the fold of change, in mean ± SEM, *n* = 3–4. (*) *p* < 0.05 against Aβ‐treated control.

Preventing Aβ‐induced mitochondrial stress has been demonstrated as a promising way against AD progression. Here, JC‐1 staining was employed to evaluate the MMP change upon Aβ treatment. Interestingly, the MMP of cultured SH‐SY5Y cells experienced a minor increase with low concentrations of Aβ (<1 μM). In contrast, high concentrations of Aβ declined MMP in both cultures, and Aβ at 10 μM was able to trigger a ~50% MMP loss (Figure 5A). In corresponding to the relieved cytotoxicity, the combined use of apigenin and BDNF markedly restored the MMP depolarization, and which was more efficient than either apigenin or BDNF applied alone (Figure 5B). The alteration of bioenergetic features of SH‐SY5Y cells was revealed by an Agilent Seahorse XFp Extracellular Flux Analyzer. Following treatment of mitochondrial inhibitors, the real‐time OCR change could reflect the bioenergetic ability of cells. The OCR profile was established (Figure 5C); this profile was similar to a previous report in SH‐SY5Y cells, suggesting that the cells had a limited spare respiratory capacity.^22^ Aβ at 10 μM significantly reduced basal respiration, ATP‐linked respiration, as well as maximal respiration (Figure 5D). BDNF at 5 ng/mL exerted a weak effect: BDNF at 15 ng/mL further strengthen the bioenergetic status of the damaged cells. As anticipated, the co‐treatment of BDNF and apigenin alleviated the mitochondrial functions, particularly in terms of maximal respiration (Figure 5D). In addition, mitochondria are the main source of ROS, which will be substantially generated because of Aβ damage. DHE and MitoSOX staining were employed to determine intracellular and mitochondrial superoxide, respectively. As shown in Figure 5E left panel, Aβ at 10 μM significantly increased the density of cells with intense DHE‐stained red nucleus, indicating the loaded DHE had been oxidized to ethidium and had integrated with nucleus DNA. In parallel, the fluorescent intensity of mitoSOX was elevated by Aβ treatment. Apigenin at 2 μM alone only exhibited weak effect in against superoxide. BDNF at 5 ng/mL was more effective in reducing mitochondrial superoxide, while a higher concentration of BDNF at 15 ng/mL efficiently reduced both superoxide levels. The combined usage of BDNF and apigenin extinguished the Aβ‐induced intracellular and mitochondrial superoxide for about 60% and 50%, respectively (Figure 5E right panel).

### 3.6 The synergistic effects of apigenin and BDNF are mediated through Trk B signaling

**FIGURE 6:**
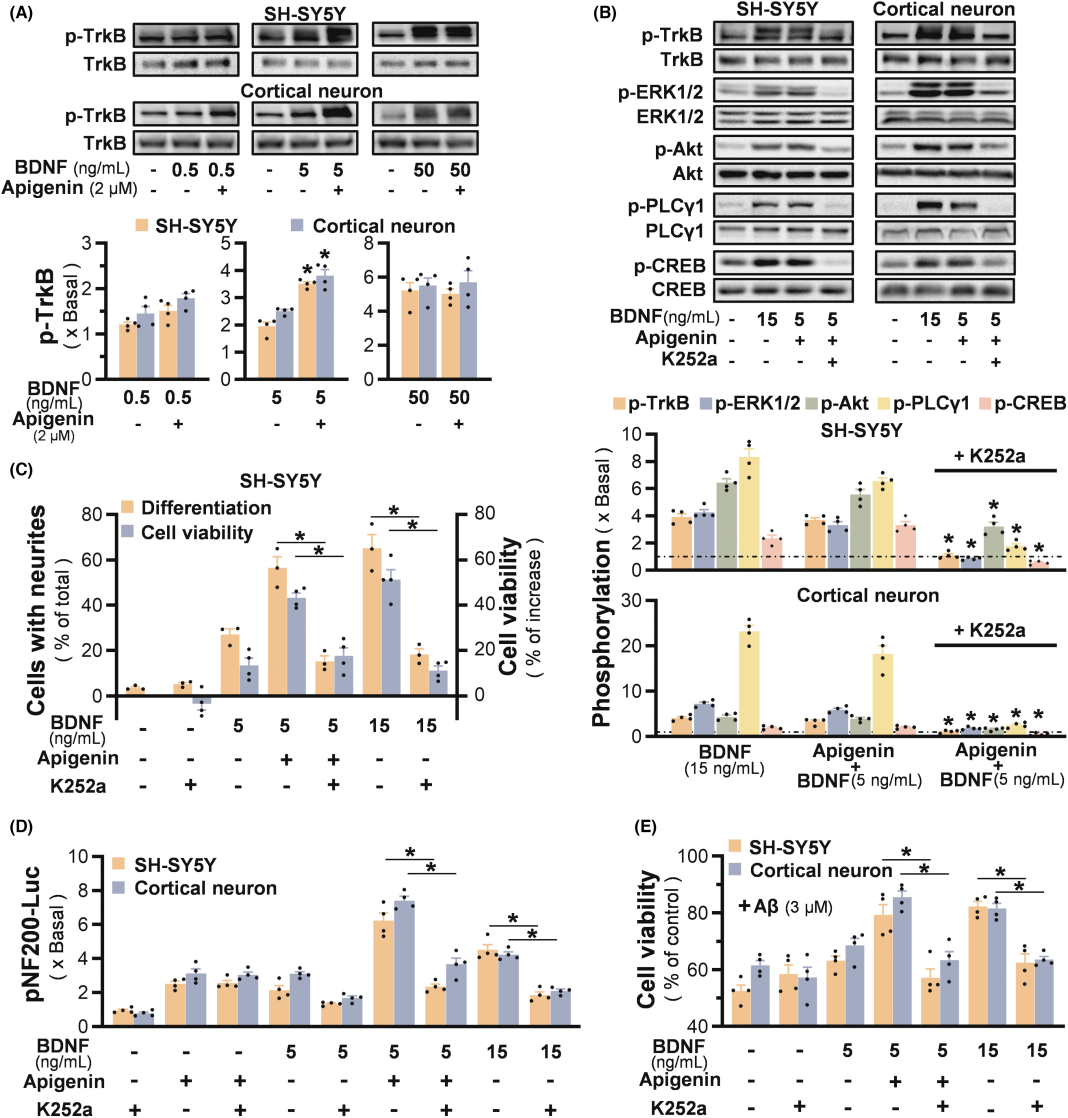
The synergistic effects of apigenin and BDNF are mediated through Trk B signaling. (A) Serum starved SH‐SY5Y cells, or cortical neurons at DIV4, were treated with BDNF for 15 min, in the presence or absence of apigenin. The triggered activation of Trk B (~140 kDa) was defined by western blot. Representative blots and the quantification result were shown. (B) Serum starved SH‐SY5Y cells, or cortical neurons at DIV4, were pretreated with 100 nM K252a for 3 h, followed by the apigenin and BDNF for 15 min. The triggered phosphorylation of Trk B (~140 kDa) as well as its downstream targets, i.e. ERK1/2 (~42/44 kDa), Akt (~60 kDa), PLCγ1 (~150 kDa), and CREB (~43 kDa) were analyzed by western blot. Representative blots (upper panel) and the quantification result (bottom panel) were shown. (C) SH‐SY5Y cells were pre‐incubated with K252a (100 nM) for 3 h, prior to treatment of BDNF, apigenin, and BDNF + apigenin in basal DMEM medium. Three days later, the percentage of cells with connected neurites was analyzed. In parallel, MTT assay was performed after 2 days of treatment. (D) Retinoic acid pre‐incubated SH‐SY5Y cells, or cortical neurons at DIV3, were transfected with pNF200‐Luc construct, and then incubated with K252a (100 nM) for 3 h, prior to 48 h treatment of apigenin and BDNF. Luciferase activity was measured in equalized by the amount of protein. (E) K252a (100 nM) was applied to retinoic acid pretreated SH‐SY5Y cells, or cultured cortical neurons at DIV4 for 3 h, followed by one hour of BDNF/apigenin pre‐incubation, as well as the 24 h of Aβ_25‐35_ (3 μM) challenge. Cell viability was measured by MTT assay. Values are expressed in the fold of change to the control, or as percentage of increase, or as percentage of total number of cells, or as percentage of control, in mean ± SEM, *n* = 3–4. (*) *p* < 0.05.

BDNF triggers Trk B receptor for its autophosphorylation and dimerization, thereby activating the receptor signaling cascades. The aforementioned synergy of BDNF and apigenin could be mediated by enhancement of Trk B receptor activation. Thus, the phosphorylation level of Trk B, stimulated by BDNF in the presence or absence of apigenin, was identified (Figure 6A). In SH‐SY5Y cells and cortical neurons, BDNF at 0.5 ng/mL did not cause a significant increase of Trk B phosphorylation, and an enhancement of the receptor phosphorylation was identified under co‐applied 2 μM apigenin. At BDNF of 5 ng/mL, apigenin further strengthened the BDNF‐evoked Trk B, exerting over ~50% increase of phosphorylating level (Figure 6A). BDNF at high dose of 50 ng/mL did not affect by the applied apigenin, probably the receptor phosphorylation was saturated under this condition. Besides, apigenin showed no effect on Trk B phosphorylation and its expression in cultured SH‐SY5Y cells, indicating that apigenin alone did not affect Trk B receptor sensitivity (Figure S2A,B). After washing out of apigenin, the application of BDNF did not show enhancement of Trk B phosphorylation (Figure S2C), suggesting the close partnership of BDNF and apigenin.

To show the receptor specificity, a Trk inhibitor K252a was utilized to validate the neurotrophic activities mediated by the synergy of BDNF and apigenin. The phosphorylation of Trk B, as well as its downstream signalings of p‐ERK, p‐Akt, p‐PLCγ, p‐CREB, was mostly blocked by applied K252a in both neuronal cultures (Figure 6B). Meanwhile, K252a substantially suppressed the BDNF/apigenin‐induced cell viability and neurite outgrowth (Figure 6C). A luciferase reporter construct, i.e., pNF200‐Luc, was employed to validate the promoter activation of NF200. BDNF at 5 ng/mL and apigenin at 2 μM induced the transcriptional activation of neurofilament by two folds (Figure 6D). The induction by apigenin was not sensitive to K252a blockage, while the BDNF‐induced pNF200‐Luc transcription was blocked by K252a. The synergy promoted the luciferase intensity, which was significantly higher than that of applied alone, attaining ~1.5 folds of 15 ng/mL BDNF. The inhibitor k252a could markedly inhibit the promoter activity, as induced by the co‐treatment or 15 ng/mL BDNF (Figure 6D). Moreover, the neuroprotective effect against Aβ was also substantially blocked by the applied K252a in both types of cultures (Figure 6E). These results indicated that the neurotrophic activities and protective effects of apigenin/BDNF were mediated by the Trk receptor.

## 4 DISCUSSION

Similar to many other flavonoids, apigenin is a phytoestrogen with weak estrogenic property. Apigenin possesses superb anti‐oxidative and anti‐inflammatory effects, exerting potential therapeutical efficacy in treating a series of health problems. Several clinical trials have been conducted to validate its bioactivity, such as prevention of skin aging, increasing blood antioxidant enzyme, reducing recurrence risk of neoplasia, as well as pain relief in migraine. The chamomile extract having high amount of apigenin has been demonstrated, clinically, in reducing the depression rating scale of patients with a primary DSM IV Axis I diagnosis of generalized anxiety disorder.^23^, ^24^ In line with neuroprotective and neurotrophic functions, as shown in the current work, the efficacy of apigenin in neurodegenerative diseases and depression has also been reported in various animal models, in which the mechanistic action is often linked to an increase of Trk‐mediated signaling, as well as an increase of BDNF level.^13^ However, extremely limited studies have been conducted in revealing the exact molecular mechanism of the apigenin‐mediated neuro‐beneficial effects.

This study highlighted a direct interaction between apigenin and BDNF, as well as their functional synergy. We hypothesize that the binding of apigenin onto BDNF could potentiate the neurotrophic activities. Indeed, the combined usage of apigenin and BDNF at low‐concentration synergistically promoted viability and neurogenesis of cultured neuronal cells. When the cultures were exposed to Aβ_25‐35_, apigenin was able to enhance the protective effects of BDNF in reducing the Aβ‐induced toxicity and mitochondrial dysfunction. Furthermore, apigenin alone could not activate the BDNF‐specific Trk B receptor, while together with BDNF, they showed synergy. The signaling specificity was validated by a Trk inhibitor, K252a, that blocked the activation of Trk receptor signaling, as well as the neurotrophic or neuroprotective properties, mediated by the co‐treatment of BDNF and apigenin.

Apigenin possesses moderate permeability to BBB. A detection of 0.42 μg/g of apigenin, estimated to be ~1.55 μM, has been detected in mouse brain tissues after a single intraperitoneal administration of apigenin at 0.4 mg/g.^25^, ^26^ In the brain, apigenin may exert its effect directly on neurons or glial cells, while simultaneously binding to BDNF and enhancing its neurotropic functions. Under pathological conditions, the activation of BDNF signaling, potentiated by apigenin, is expected to relieve the patients from neurological diseases and emotional disorders. Besides, miRNAs have been proposed to play an important role in neurogenesis and synaptic plasticity, and the dysregulation of miRNAs is involved in pathogenesis of depression.^27^ The level of BDNF has been shown to correlate with the expressions of depression‐associated miRNAs, such as miR‐30a. The regulation of these miRNAs by apigenin has also been revealed.^28^ In addition, the abnormalities of immune system have been widely documented to contribute significantly to patients suffering from depression.^29^ The pro‐inflammatory mediators could lead to disordered reuptake and synthesis of neurotransmitters, reduced neurogenesis of hippocampus, as well as the hyperactivated hypothalamic–pituitary–adrenal axis. In line to this notion, apigenin may work with BDNF in resisting neuroinflammation; because both apigenin and BDNF have been demonstrated to modulate inflammatory responses.^13^, ^30^

Flavonoid is a popular class of compounds in searching for candidate drugs mimicking the function of neurotrophic factors. Some of the flavonoids have been reported to induce differentiation, or neurite outgrowth, of cultured neuronal cells, and these effects could be explained by the activations of Trk receptor, PI3K‐Akt, ERK1/2‐CREB, estrogen receptor, and calcium signaling.^12^ In different cell models, apigenin was able to promote cell differentiation or neurogenesis in neuronal cell cultures.^31^, ^32^, ^33^, ^34^ However, flavonoids are not able to induce the differentiation as significant as that of neurotrophic factors, because neurite extension is a combinatorial process having interplay of intracellular signals.^35^, ^36^ In general, apigenin did not show better specificity as compared to other flavonoids in inducing the neuronal differentiation.^20^, ^37^ Here, apigenin alone exhibited very limited neurotrophic activity, and therefore, the degree of flavonoid‐meditated neurotrophic activities, e.g., differentiation, could depend on the cell types.

Among the neurotrophic factors, NGF is commonly considered to have synergy with candidate drugs to have higher activity of neurotrophic functions. The flavonoid‐induced neuronal differentiation could get potentiated when co‐applying with a small amount of NGF.^37^, ^38^ In cultured PC12 cells, the binding of flavonoids onto estrogen/progesterone receptor, or Na+/K+/Cl‐ co‐transporter, is known to enhance the NGF function.^17^, ^34^, ^39^, ^40^ In addition, the activation of Trk downstream targets, mediated by flavonoids, may lead to a stronger NGF activity.^38^ Here, we have evaluated other flavonoids, but none of them showed synergy with BDNF as good as that of apigenin in SH‐SY5Y cells (data not shown). Thus, the direct interaction of apigenin with BDNF should account for the potentiation of neuronal differentiation.

Mature BDNF is a noncovalently associated dimer and is structurally similar to other neurotrophic factors. In our molecular docking result, the residues 8–116 of BDNF (PDB:1B8M) were recognized as a domain binding to Trk B receptor. Several H‐bonds were established between apigenin and amino acid residues, such as Glu9, Ser11, and Met61, which anchored the accommodation of the ligand at the binding domain. Here, we hypothesize that the apigenin/BDNF interaction could increase the binding affinity of BDNF to Trk B receptor, which thereby leads to the enhancement of downstream signaling. However, the low solubility of flavonoid raises the question of whether the oral availability of apigenin could support its potentiating effects in vivo. We supposed the apigenin's metabolites may possess similar interaction with BDNF as apigenin does. In addition, the binding of flavonoids onto BDNF does not guarantee the functional activation. Among several other flavonoids with a good docking score to BDNF, only alpinetin showed a weak synergism with BDNF in inducing differentiation of SH‐SY5Y cells.

Today, nutritionists have been dedicated to finding ways of increasing endogenous BDNF levels. Useful tips, such as regular exercise, everyday good sleep, intermittent fasting, as well as having more plant‐derived foods, may help to secrete more BDNF from the brain and to promote neurogenesis.^41^, ^42^, ^43^ The search for therapeutic products from natural sources, e.g., utilizing effective chemicals derived from plants, is considered as an effective approach. With this regard, the take‐home message here is the inclusion of apigenin into our diet could boost the BDNF function, thereby benefiting our brain health. In current nutraceutical market, the products containing apigenin are very popular, having a recommended daily intake of ~100 mg. In general, the botanic resources of apigenin are easy to get, e.g., celery, parsley, chamomile; these vegetables contain high amount of apigenin and its conjugated derivatives.^44^ Moreover, the herbal extract forms of apigenin tend to be better absorbed by human body than the pure chemical, hence, it is recommended to intake the semi‐purified apigenin extract to achieve its maximal efficacy.^45^, ^46^

## AUTHOR CONTRIBUTIONS

Participated in research design: Tsim, Gao. Conducted experiments: Gao, Xia, and Lin. Performed data analysis: Gao, Xia, and Dong. Wrote or contributed to drafting the manuscript: Tsim, Gao. Editing and approval of final manuscript: All the authors.

## FUNDING INFORMATION

This work is supported by The Key‐Area Research and Development Program of Guangdong Province (File no. 2020B1111110006); Special project of Foshan University of science and technology in 2019 (FSUST19‐SRI10); GBA Institute of Collaborate Innovation (GICI‐022); Shenzhen Science and Technology Innovation Committee (ZDSYS201707281432317, JCYJ20170413173747440, JCYJ20180306174903174), Zhongshan Municipal Bureau of Science and Technology (ZSST20SC03); Guangzhou Science and Technology Committee Research Grant (GZSTI16SC02, GZSTI17SC02); Hong Kong RGC Theme‐based Research Scheme (T13‐605/18‐W); and Hong Kong Innovation Technology Fund (PRP/073/20FX, MHP/004/21, ITCPD/17–9).

## CONFLICT OF INTEREST STATEMENT

The authors declare that they have no competing interests.

## Supporting information


Figure S1.

Figure S2.

## Data Availability

The data that support the findings of this study are available from the corresponding author upon reasonable request.
