# Supplementary figures and images for: The neurotrophic activities of brain‐derived neurotrophic factor are potentiated by binding with apigenin, a common flavone in vegetables, in stimulating the receptor signaling
Source: CNS Neurosci Ther. 2023 Apr 26;29(10):2787–99. doi: 10.1111/cns.14230 (PMC10493664; doi:10.1111/cns.14230)

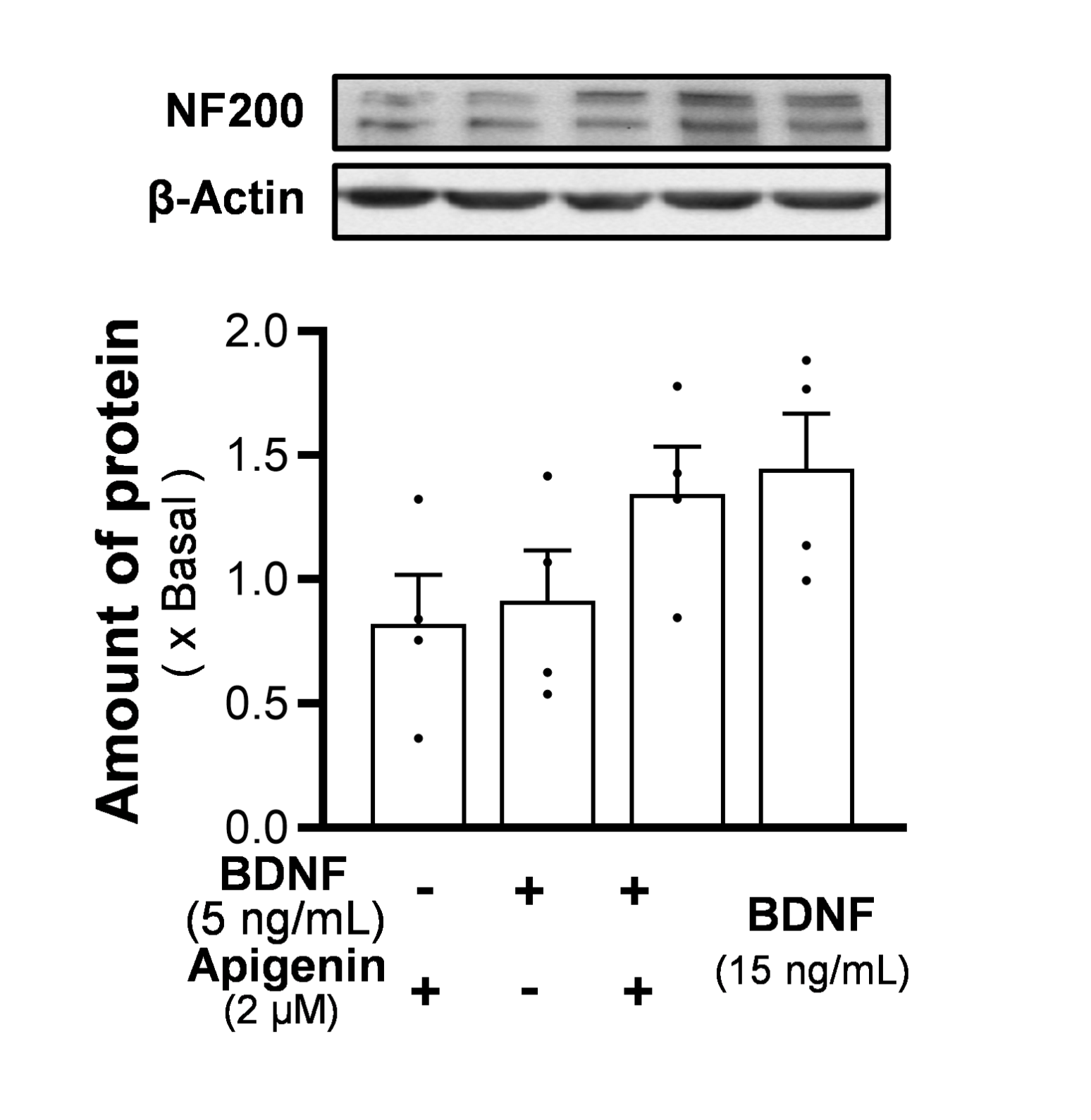

Supplement: Supplementary file 1 — Figure S1. [file CNS-29-2787-s002.tif]

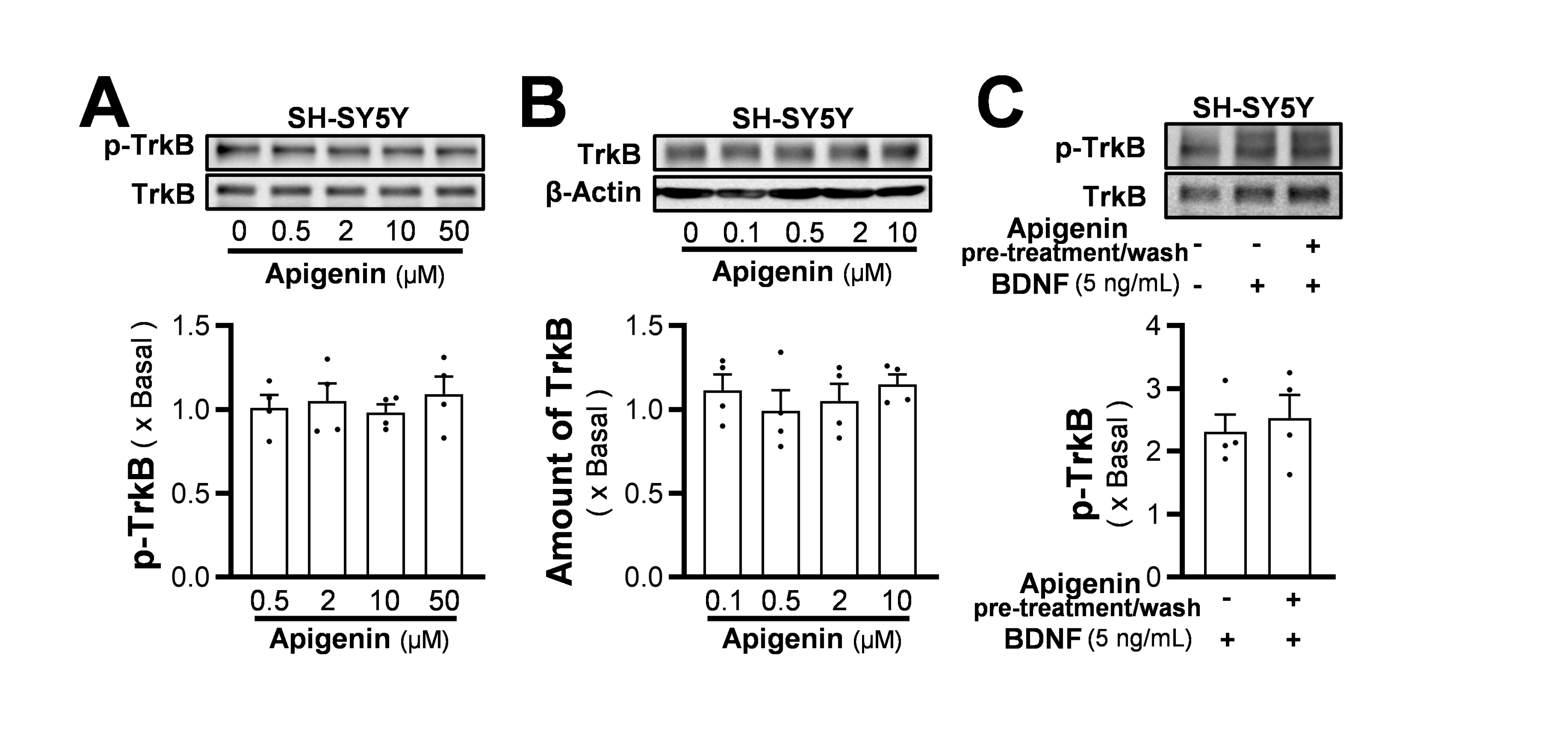

Supplement: Supplementary file 2 — Figure S2. [file CNS-29-2787-s001.tif]
